# The Development and Testing of KNACC: Knowledgeable Nursing Assistants as Creative Caregivers

**DOI:** 10.1093/geroni/igaa057.2346

**Published:** 2020-12-16

**Authors:** Jacqueline Eaton, Kristin Cloyes, Brooke Paulsen, Connie Madden, Lee Ellington

**Affiliations:** 1 University of Utah, Salt Lake City, Utah, United States; 2 University of Utah, Portland, Oregon, United States

## Abstract

Nursing assistants (NAs) provide 80% of direct care in long-term care settings, yet are seldom viewed as skilled professionals. Empowering NAs is linked to improved resident outcomes. In this study, we collaborate with NAs to adapt and test the feasibility and acceptability of arts-based creative caregiving techniques (CCG) for use in long-term care. We held a series of focus groups (n=14) to adapt, refine, and enhance usability. We then evaluated implementation in two waves of testing (n=8). Those working in memory care units were more likely to use all techniques, while those working in rehabilitation were more hesitant to implement. Participants reported using CCG to distract upset residents. Family members were excited about implementation, and NAs not participating wanted to learn CCG. Nursing assistants have the potential to become experts in creative caregiving but may require in-depth training to improve use.

